# The correlation between clinical, nuclear and histologic findings in a patient with Von Recklinghausen's disease

**DOI:** 10.1186/1477-7819-5-130

**Published:** 2007-11-12

**Authors:** Justus-Martijn Brinkman, Johannes L Bron, Paul IJM Wuisman, Paul J van Diest, Emile FI Comans, Carla FM Molthoff

**Affiliations:** 1 Department of Orthopaedic Surgery, VU University Medical Center, Amsterdam, The Netherlands; 2 Department of Pathology, VU University Medical Center, Amsterdam, The Netherlands, currently: Department of Pathology, University Medical Center, Utrecht; 3 Department of Nuclear Medicine & PET Research, VU University Medical Center, Amsterdam, The Netherlands; 4 Department of Surgery, Deventer Ziekenhuis, Deventer, The Netherlands

## Abstract

**Background:**

Malignant peripheral nerve sheath tumours (MPNST) are known to develop in patients with Neurofibromatosis type I (NF1) resulting in a decreased overall survival. The association between NF1 and the development of such MPNST has been investigated in detail. The biological behaviour however of multiple disseminated neurofibromas in patients with NF1 and the risk factors for malignant transformation remain unknown. Clinical signs are unreliable and additional imaging techniques are therefore required. Of such, positron emission tomography using [^18^F]-2-fluoro-2-deoxy-D-glucose (^18^FDG PET) is used to detect malignant changes in neurofibromas.

**Case presentation:**

A case is presented of a patient suffering from NF1 with clinical signs of malignant change and accumulation of ^18^FDG in multiple neurofibromas. Histopathological examination of 20 lesions however, did not reveal any malignant features. There was no statistically significant relation between^18^FDG accumulation and malignant change, but rather with pain, size and growth.

**Conclusion:**

This case adds to the knowledge of the diverse biological behaviour of neurofibromas in patients with NF1

## Background

Neurofibromatosis type I (NF1) is an autosomal dominant multisystem disorder with an incidence of approximately 1 in every 3500 births [1-3]. The classical features of NF1 are *café au lait *spots, neurofibromas, iris Lisch nodules and skin fold freckling [3,4]. The neurofibromas arise from peripheral nerve branches or sheaths of peripheral nerve fibers and are derived from Schwann cells or pluripotent cells of neural crest origin [5]. The lesions can occur at virtually any site in the body and are generally divided in nodular plexiform, diffuse plexiform, subcutaneous and cutaneous types (14). Patients with NF1 have a decreased overall survival due to the development of malignant peripheral nerve sheath tumours (MPNST). MPNST arise from plexiform neurofibromas in approximately 10% of the patients [3,3,5-7]. MPNST may metastasize widely and are therefore associated with a poor prognosis.

Diagnosis of a MPNST in patients with NF1 is based on clinical, radiological and histological investigation. Rapid growth, pain and neurological impairment are symptoms associated with malignant transformation, however, the symptoms are not specific and may also occur in benign lesions [5,6]. Magnetic Resonance Imaging (MRI) accurately reveals the site and extent of the lesion, but cannot differentiate between benign and malignant lesions [8,9]. Histopathological examination of biopsy material is necessary, but even this is not always conclusive since the retrieved specimens may not represent the overall characteristics of the tumour. Areas of high-grade activity between larger areas of benign cells may be missed due to sampling error [10]. Therefore, various radionuclide imaging techniques have been described to detect the development of MPNST in patients with NF1 [3,11,12]. Because sarcomas have high glucose metabolic rates ^18^fluorodeoxyglucose (^18^FDG) positron emission tomography (PET) scanning is well established [1,4-6,13].

The presented case describes the correlation between clinical, radiological, FDG-PET scanning and histopathological findings in a patient with NF1, clinically suspected of developing MPNST.

## Case presentation

**Figure 1:**
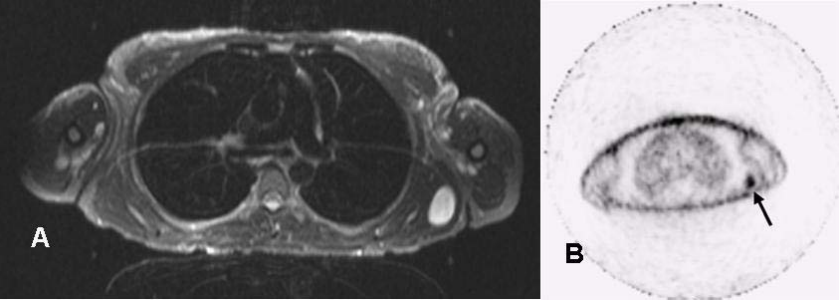
Magnetic resonance imaging A) Transaxial MRI scan (STIR, 5 mm slice thickness, left) B) Corresponding ^18^FDG PET scan (5 mm slice thickness, right). Increased FDG uptake is seen in a subcutaneous lesion in the left shoulder region (arrow).

**Figure 2:**
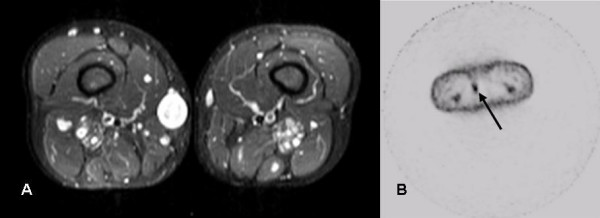
A) Transaxial MRI scan (STIR, 5 mm slice thickness, left) and B) Corresponding FDG-PET scan (5 mm slice thickness, right). Increased FDG uptake is visible in a subcutaneous lesion located medially in the right upper leg (arrow).

A 39-year old woman, known with NF1 and Sickle cell anaemia, was referred to our hospital with complaints of pain and notable growth of multiple neurofibromas (Table 1). At examination, the patient had multiple cutaneously and subcutaneously located neurofibromas, most notably at the following locations: left ankle, left lower leg, right upper leg, right buttock (two) and right hip, varying in size from four to eight centimetres (Table 1). MRI studies of the suspected area's showed well-defined, circumscribed lesions having a homogenous aspect without signs of invasion of surrounding structures. The signal intensity was low on T1, moderate on T2 and high on STIR. Thus, the MRI-studies were consistent with multiple, complete benign appearing neurofibromas. ^18^FDG PET-scanning however, showed multiple sites of increased ^18^FDG accumulation (Figure 1A and 1B). The corresponding lesions were clearly detectable on the MRI-studies and palpable on clinical examination. Therefore, ten neurofibromas were selected for surgical resection (I-X, Table 1), including four lesions with increased ^18^FDG accumulation. Seven months post-operative, ^18^FDG PET-studies showed no increased ^18^FDG accumulation in new areas, nor in the neurofibromas left *in situ*, nor at the sites of resected neurofibroma. One year postoperative however, the patient started to complain about pain and discomfort from neurofibromas that previously had been asymptomatic (XI-XX, Table 1). On clinical examination most notable locations were: dorsal side of the left and right wrist, right scapula and left shoulder (previously documented), varying in size on palpation from two to eight centimetres. ^18^FDG PET was performed revealing multiple new as well as previously documented sites of increased ^18^FDG accumulation (Figure 2, Table 1). Again, ten neurofibromas (XI-XX) are selected for surgical resection. The surgical interventions were both uneventful.

**Table 1:** Details for each neurofibroma resected at the first and second operation

	**Neurofibroma**	**Location**	**Type**	**Pain**	**Growth**	**Size (cm)**	**FDG score**	**Amount**	**Pseudo capsule**	**Glut-1**	**HK II**
Operation 1	I	R buttock	NP	+	+	2	+	4	-	-	++
	II	R buttock 2	SC	+	+	4	+	3	+	-	+
	III	R flank	SC	-	-	4	-	1	+	-	+
	IV	Abdomen	NP	-	-	1.5	-	1	-	-	+
	V	L upper arm	C	-	-	1	-	1	-	-	++
	VI	Pubic area	C	-	-	1	-	1	-	-	++
	VII	L calf	C	+	-	1	+	3	-	-	+
	VIII	R lower leg	DP	-	-	1.5	-	1	-	-	++
	IX	L ankle	SC	+	+	4	+	4	+	-	+
	X	L knee	SC	-	-	1	-	1	-	-	-
Operation 2	I	Sternum	DP	-	-	1.5	-	1	-	-	+
	II	R wrist rad	SC	-	-	4	-	1	-	-	+++
	III	R wrist uln	NP	+	+	5	+	3	+	-	++
	IV	L breast	DP	-	-	2.5	-	1	-	-	+
	V	R upper leg	SC	-	-	6	+	3	+	-	+++
	VI	L wrist prox	C	-	-	3	+	4	-	-	-
	VII	L wrist dist	C	-	-	8	+	3	+	-	++
	VIII	L arm pit	SC	-	-	5	+	4	+	-	++
	IX	R scapula	SC	+	-	3	-	1	-	-	+
	X	Lower back	C	-	-	1	-	1	-	-	+++

R = right, L = left, NP = nodular plexiform, SC = subcutaneous, C = cutaneous, DP = diffuse plexiform, Pain: + = pain, – = no pain, Growth: + = growth, – = no growth, Size: size on MRI in centimetres (cm) FDG uptake: + = accumulation of ^18^FDG on PET scanning, – = no uptake, Amount (see text): semi quantitative measurement of ^18^FDG uptake, Pseudo C: + formation of a pseudo capsule, – = no capsule, Glut-1: + = expression of Glut-1, – = no expression, HKII: + = semi quantitative measurement of HKII expression, – = no expression.

### FDG PET scan

The PET-studies were performed using an ECAT EXACT HR+ scanner (Siemens/CTI) after injection of 370 MBq of [^18^F]-2-fluoro-2-deoxy-D-glucose (^18^FDG) and 2D-images are obtained 60 minutes after injection. All scans were corrected for decay, scatter and randoms and were reconstructed using ordered subset expectation maximization (OSEM) with 2 iterations and 16 subsets followed by post smoothing of the reconstructed images using a 5 mm FWHM Gaussian filter. No attenuation correction was applied. Visual analysis of the PET scans was performed with regard to size and intensity of ^18^FDG accumulation, using a 4-point scale; 1: no uptake, 2: sparse uptake, 3: moderate uptake, and 4: high uptake.

### Histology and immunohistochemistry

**Figure 3:**
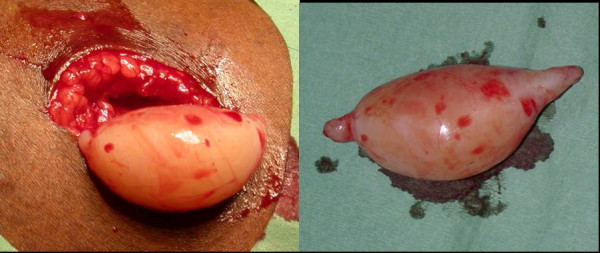
A well defined, circumscribed neurofibroma, without signs of macroscopic invasion of surrounding structures, located on the patients right upper leg, which showed ^18^FDG accumulation on PET scanning.

In total, twenty neurofibromas were resected during the two separate surgical interventions (ten each) and the specimens (figure 3) were analyzed both, histopathologically and immunohistochemically. All specimens were fixed in neutral 4% buffered formaldehyde for at least 24 hours. Immunohistochemical analysis was performed on 4 μm thick tissue sections. After deparaffination and rehydration, endogenous peroxidase activity was blocked. After antigen retrieval, a cooling-off period preceeds the incubation of the antibodies for the glucose transporter protein (Glut-1; DAKO, Glostrup, Denmark) and hexokinase II (HK II; Chemicon, UK). Specific binding was detected with a biotinylated swine-anti-rabbit-antibody (DAKO) and an avidin-biotin complex (DAKO). Staining was developed with diamminobenzidine and counterstained with haematoxylin/eosin. Intensity of staining was categorized as negative (-), weak (+), positive (++) and strongly positive (+++). Negative (obtained by omission of the primary antibody) and positive controls were used throughout. For Glut-1 staining an internal control was staining of erythrocytes (positive for glucosetransporter-1).

### Statistics

Statistical analysis of correlation between complaints (pain and growth), size, tumour type, semi-quantitative measurement of ^18^FDG accumulation and morphologic (pseudo capsule formation) and immunohistochemical characteristics (Glut 1 and HK II expression) was performed using Spearman's Rank Correlation Coefficient. SPSS software (Release v12.0, SPSS Inc, Chicago, Ill, USA) was used for the statistical analyses. P-values < 0.05 were designated significant.

## Results

Macroscopically, the specimens of both surgical interventions show features of completely benign neurofibromas. All specimens show well defined, circumscribed neurofibroma without signs of macroscopic invasion of surrounding structures (Figure 3). Microscopically, all neurofibromas show the same morphological characteristics including spindle cell proliferations with small, elongated, hyperchromatic nuclei. In neurofibromas II, III, IX, XIII, XIV, XVII and XVIII a slightly more expansive growth pattern can be observed with increased cellularity and formation of a pseudo-capsule. The surrounding pseudo capsule is in fact the perineurium and indicates an origin of the neurofibroma within a nerve. From the examined lesions, 6 were plexiform neurofibroma's and the remainder of the lesions were either cutaneous or subcutaneous according to Riccardi [14]. Although there is a slight variation in cellularity between different fibromas, no nuclear atypia and no mitosis are seen.

**Figure 4:**
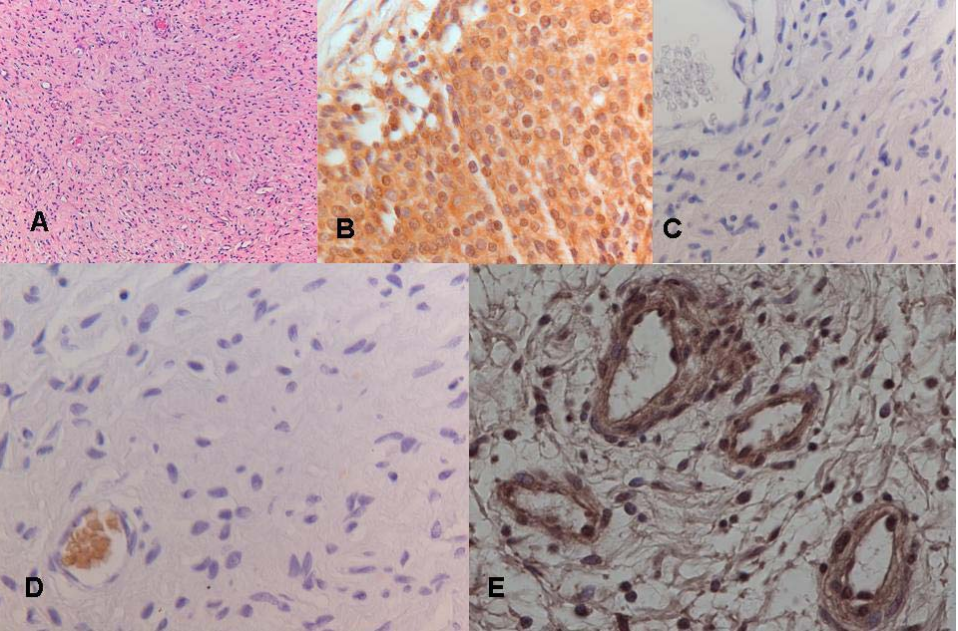
Histological and Immunohistochemical analysis of the same neurofibroma. A) overview of the tumour stained with haematoxylin (magnification 100×); B) positive control for HK II (staining of a cervical carcinoma section); C) negative control for Glut-1 and HK II; D) Glut-1 staining (negative) with positively stained erythrocytes (internal positive control); E) HK II staining (positive) (B-E: magnification 400×)

Immunohistochemically, tumor cells stain all positive for HK II with varying intensity, sporadically positive for Ki67 and negative for Glut-1 (Table 1, Figure 4). Expression of Glut-1 is regulated by oncogenes and growth factors, and causes increased activity of glycolytic enzymes [13,15]. Histopathological diagnosis of all 20 resected tumours results in neurofibroma without signs of malignant transformation. Six neurofibromas with increased ^18^FDG accumulation show more expansive growth accompanied with the formation of a pseudo capsule in three lesions (Table 1). One neurofibroma without ^18^FDG accumulation shows the formation of a pseudo capsule, whereas 3 neurofibromas with accumulation of ^18^FDG lack a pseudo capsule (Table 1). There is a statistically significant correlation between; pain and growth (p < 0.01), size and the formation of a pseudo capsule (p < 0.01), and between pain, growth and size and ^18^FDG accumulation (p < 0.05).

## Discussion

MPNST account for 10% of all soft tissue sarcomas with half of these malignancies arising in patients with NF1 [16]. The overall prognosis of MPNST is poor, with a 5-year survival rate of only 20 % [4,17,18]. NF1 patients have a higher incidence of MPNST at an early age [2,19,20]. There is a peak incidence between 25 and 30 years of age, compared to between 40 and 60 years for MPNST without NF1 [2,16,17]. In the present study, the patient was diagnosed with neurofibromatosis and had clinical signs suspected for malignant transformation. In addition, accumulation of ^18^FDG was seen on PET scanning in multiple neurofibromas. We showed that neither clinical suspicion on MPNST, nor the presence of ^18^FDG accumulation, nor both features combined are certain parameters for malignancy. Histopathologic analysis of 20 neurofibromas divided over two separate occasions revealed completely benign lesions. Immunohistochemical analysis showed hexokinase expression in all resected lesions. Previous reports have documented ^18^FDG accumulation in histopathologically benign neurofibromas [1,21]. In this case however, all neurofibromas with ^18^FDG accumulation turned out to be benign. To our knowledge this has not been documented previously. Statistically, pain and growth, size, the formation of a pseudo capsule, and ^18^FDG accumulation were associated. There was no statistically significant association between ^18^FDG accumulation and tumour type or the intensity of hexokinase expression.

Pain associated with a mass, and the presence of either subcutaneous or cutaneous neurofibromas, has been reported by King *et al*., [2] to be the greatest risk factor for MPNST in patients with NF1. The presence of either subcutaneous or cutaneous neurofibromas is of limited value however, because of their high incidence in NF1 patients. Furthermore, these investigators reported on 8 patients with MPNST without pre-existing clinically detectable plexiform neurofibroma [2]. Ducatman *et al*., [17] reported 81% of MPNST to be associated with pre-existing neurofibromatous tissue on pathological examination. Both findings suggest that patients who lack clinically detectable or symptomatic neurofibroma are not exempt from the development of an MPNST.

Radionuclide imaging has been used to detect sarcomatous changes in neurofibroma. Lee *et al*., [3] reported increased Ga-67, Th-201 and Tc-99 m (V) DMSA uptake in a MPNST (or neurofibrosarcoma) but not in benign neurofibromas present in the same patient. Solomon *et al*., [6] reported on a single mass in a patient with increased glucose metabolism seen on a pre-operative PET scan, corresponding with malignant transformation of a neurofibroma at pathological examination. Ferner *et al*., [1] performed a qualitative and quantitative assessment of ^18^FDG accumulation in 18 patients with a known history of NF1 with a clinical suspicion of malignant transformation of neurofibroma. In ten patients, histological confirmation was obtained of the suspected lesions based on ^18^FDG PET. No false negatives and two false positives were reported and it was concluded that ^18^FDG PET could be a useful method of identifying malignant change in neurofibroma in NF1 patients. Cardona *et al*., [4] evaluated the nature of 25 neurogenic tumours suspected of malignancy, in patients with NF1, using ^18^FDG PET. Three-dimensional qualitative and quantitative assessment of FDG-PET scanning was compared to histological reports; no histopathologically malignant tumours were classified benign, but three benign tumours had been rated malignant. Standard uptake value (SUV) was compared to histopathological diagnosis; the SUV was significantly higher in MPNST at a cut off-value of 1.8 SUV. Cordana *et al*., recommended the use of ^18^FDG PET scanning in the follow-up of patients with NF1 and that those lesions with a SUV of 1.8 or more should be resected. Recently, Brenner *et al*., showed FDG PET scanning to be a stronger parameter for the prediction of survival in patients with MPNST than histopathological grading [22].

Malignant cells have an increased glucose uptake and metabolism demonstrated both *in vitro *and *in vivo *[15]. In various cancers, such as tumours of the oesophagus, colon, pancreas, stomach, brain and breast, the major glucose transporters protein (Glut-1) has been reported to be up regulated [13,15]. To our knowledge this has not been investigated in neurofibrosarcoma. Hexokinase is the first glycolytic enzyme to phosphorylate glucose and FDG intracellularly [13,15]. Increased expression of hexokinases in breast and lung cancer has been described, but not in other tumours [13]. In the current case immunohistochemical analysis was performed for the presence of Glut-1 and hexokinase expression; there was no Glut-1 expression and expression of hexokinase varied in intensity. Hence, no association could be found between the immunohistochemical results and the 18FDG PET scans.

## Conclusion

We conclude that in this case there was no correlation between ^18^FDG accumulation and malignancy, but rather with pain, size and growth of the neurofibromas. The findings in this patient with neurofibomatosis do not support the use of FDG PET in the setting of screening for malignant transformation. However, FDG PET may be a useful tool to rule out malignancy in patients with a clinical suspicion for malignant transformation in lesions that are difficult for surgical biopsy. This case adds to the knowledge of the diverse biological behaviour of neurofibromas in patients with NF1.

## Competing interests

The author(s) declare that they have no competing interests.

## Authors' contributions

**J-MB **conceived the idea for the study, participated in its design and coordination and wrote the first draft of the manuscript.

**JLB **conducted the literature review and helped to draft the manuscript.

**CFMM **and **EFIC **collected and analysed the data of the ^18^FDG PET scans and helped to write the concerning passages in the manuscript.

**PJD **and **CFMM **collected and analysed the histopathological data.

**PIJMW **supervised in the study and helped to draft the manuscript.

All authors read and approved the final version of the manuscript.
